# Optimization of the *Lactococcus lactis *nisin-controlled gene expression system NICE for industrial applications

**DOI:** 10.1186/1475-2859-4-16

**Published:** 2005-05-30

**Authors:** Igor Mierau, Kees Olieman, James Mond, Eddy J Smid

**Affiliations:** 1 NIZO food research, P.O. Box 20, 6710 BA EDE, The Netherlands; 2 Biosynexus Inc., 9119 Gaither Road, Gaithersburg, MD 20877, USA

## Abstract

**Background:**

The nisin-controlled gene expression system NICE of *Lactococcus lactis *is one of the most widely used expression systems in Gram-positive bacteria. Despite its widespread use, no optimization of the culture conditions and nisin induction has been carried out to obtain maximum yields. As a model system induced production of lysostaphin, an antibacterial protein (mainly against *Staphylococcus aureus*) produced by *S. simulans *biovar. Staphylolyticus, was used. Three main areas need optimization for maximum yields: cell density, nisin-controlled induction and protein production, and parameters specific for the target-protein.

**Results:**

In a series of pH-controlled fermentations the following parameters were optimized: pH of the culture, use of NaOH or NH_4_OH as neutralizing agent, the addition of zinc and phosphate, the fermentation temperature, the time point of induction (cell density of the culture), the amount of nisin added for induction and the amount of three basic medium components, i.e. yeast extract, peptone and lactose. For each culture growth and lysostaphin production was followed. Lysostaphin production yields depended on all parameters that were varied. In the course of the optimization a three-fold increase in lysostaphin yield was achieved from 100 mg/l to 300 mg/l.

**Conclusion:**

Protein production with the NICE gene expression system in *L. lactis *strongly depends on the medium composition, the fermentation parameters and the amount of nisin added for induction. Careful optimization of key parameters lead to a significant increase in the yield of the target protein.

## Background

*Lactococcus lactis *is a Gram-positive lactic acid bacterium that is widely used in food fermentations, such as in cheese and butter production. In the last two decades the physiology and genetics of this bacterium have been thoroughly studied [1,2]. At present the genome sequences of several strains of *L. lactis *have been elucidated, leading to an acceleration and integration of our knowledge of these bacteria [3-6]. Because of its genetic accessibility and because it is easy to handle, *L. lactis*, in addition to its traditional applications, has been extensively developed and used for the expression of heterologous genes, and has become one of the most used Gram-positive gene expression hosts. Table 1 gives an overview of the wide range of applications that involve *L. lactis *as host bacterium.

**Table 1:** Overview of various applications of the NICE system

**Application area**	**Application**	**Reference**
Expression of homologous genes	Aminopeptidase N	[7]
	ATPase system (8 gene operon)	[28]
	Aminoacylase	[29]
	Cluster of genes encoding folate biosynthesis	[19]
Expression of heterologous genes of Gram^+ ^and Gram^- ^bacteria	NADH oxidase of *Streptococcus mutans*	[30]
	Fructose bisphosphatase of *Escherichia coli*	[31]
	Green fluorescent protein of *Aequoria victoria*	[32]
	Lysostaphin of *Staphylococcus simulans *biovar. Staphylolyticus	[15]
Protein secretion	Lipase of *Staphylococcus hyicus*	[33]
	Bovine beta-lactoglobulin	[34]
Membrane proteins: prokaryotic and eukaryotic	Multidrug transporter of *Lactococcus lactis*	
	Xylidose transporter of *Lactobacillus pentosus*	
	ATP/ADP translocator of *Rickettsia prowazekii*	Review [35]
	KDEL receptor of *Homo sapiens*	
	Mitochondrial carriers of *Saccharomyces cerevisiae*	
Cloning of toxic genes	Lysis cassette of the virulent phage us3 of	[26]
	*Lactococcus lactis*	
	Autolysin gene of *Leuconostoc citreum*	[36]
Bacterial antigens	Antigen L7/12 of *Brucella abortus*	[13, 37]
	C subunit of tetanus toxin (TTFC) of *Clostridium tetani*	[38]
Viral antigens	Non-structural protein 4 of bovine rotavirus	[13, 39]
Cytokines	Interleukin 12 of *Homo sapiens*	[40]
Industrial-scale application	Lysostaphin of *Staphylococcus simulans *biovar. Staphylolyticus	[15]

**Figure 1:**
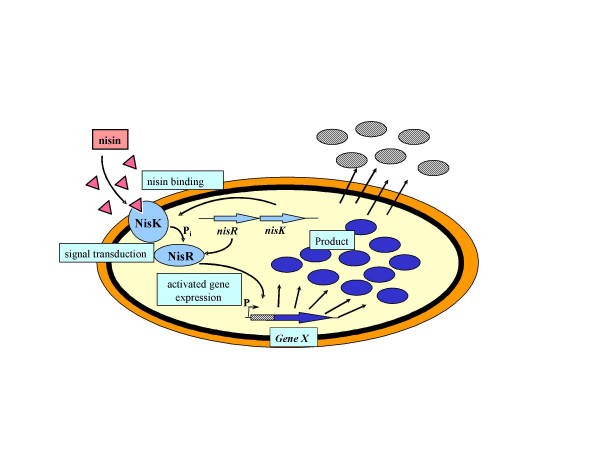
**Schematic overview of the NICE system, its components and its function. **NisK and NisR are the sensor protein and the response regulator, respectively. The product of the expressed gene can either accumulate in the cell or be secreted into the extracellular medium depending on the presence of a signal sequence in the construct.

Regulated gene expression can be of critical importance in achieving high yields of proteins. This is either important for the expression of genes that form toxic products for the cell (see Table 1) or for the conservation of energy for producing biomass prior to directed overproduction of the protein of interest. The most commonly used regulated expression system of Gram positive bacteria is the NIsin Controlled gene Expression system NICE of *L. lactis *[7]. Sub-toxic amounts of nisin in the ng/mL range are sufficient to fully activate the otherwise tightly closed promoter [8]. In the natural situation nisin binds to the receptor NisK. Subsequently NisK activates NisR by phosphorylation and the activated NisR induces the nisin operon at the nisin A promoter [9]. To exploit this system for gene expression, the genes of the receptor protein and the response regulator – *nisK *and *nisR *– have been isolated and placed on the chromosome of a suitable host strain. Furthermore, the nisin A promoter has been isolated and placed on a plasmid. When a gene is cloned down-stream of this promoter and the construct is placed in a *nisRK *strain, expression can be activated by the addition of nisin (Figure 1) [7]. Depending on the presence of a signal sequence the product is either accumulated inside the cell or secreted into the medium (see Table 1).

*Escherichia coli *is, at present, the dominant prokaryotic system for industrial gene expression. This is due to high yields, ease in genetic handling, long-term experience and extensive documentation with the US Food and Drug Administration and other regulatory bodies. However, there are also various disadvantages, such as the formation of endotoxins, the formation of inclusion bodies, the presence of two membranes, which hampers secretion, and the relatively complicated aerobic fermentation [10,11]. *L. lactis*, on the other hand, has a number of properties that make this bacterium an interesting alternative candidate for large-scale gene expression: the bacterium is food grade (used in food production for thousands of years), it is used at very large scales, and plasmid selection mechanisms are available that are food grade and self-cloning (e.g. growth on lactose) [12]. Furthermore, no endotoxins or inclusion bodies are formed and sophisticated genetic tools enable easy genetic handling [13,14]. Finally, simple, non-aerated fermentation makes direct scale-up from 1-L scale to 1000-L scale possible [2,14]. Recently, we have demonstrated in our laboratory that nisin controlled gene expression can be effectively used in 3000-L scale fermentations [15].

The NICE system has been used in a multitude of laboratory applications (Table 1), in which gene expression is often performed in acidifying cultures. The drawback of this culture type is its low final cell density due to medium acidification by lactic acid. pH-controlled fermentations, on the other hand, result in at least five-fold higher cell densities and thus higher biomass yields. However, no systematic optimization of the induction and expression of the NICE system under pH-regulated conditions has yet been performed. Three main areas need optimization for maximum yields: cell density of the culture, nisin-controlled induction and protein production, and parameters specific for the target-protein.

Lysostaphin is a 25 kD antibacterial protein produced by *Staphylococcus simulans *biovar. Staphylolyticus [16] and mainly used against multiple antibiotic resistant *S. aureus *[17,18]. In this paper we demonstrate, with lysostaphin as a model, that careful optimization of key parameters of the induction and production process can lead to an at least three-fold increase of the fermentation yield from 100 mg/L to 300 mg/L lysostaphin.

## Results

### Introduction

At present, protocols for nisin-induced gene expression only exist for acidifying batch cultures (see e.g. [7,19]) but not for pH-controlled batch cultures. Therefore, a protocol that was previously developed at our laboratory for large-scale lysostaphin production [15] was used as a starting point. The cultivation and induction conditions were as follows: pH controlled (using NaOH) growth at pH 6.5, growth temperature 30°C, inoculum 1%, induction at OD_600 _= 1 (= 0.3 g/L cell dry weight [20]) with 10 ng/mL nisin, and harvest after 6 h. The initial medium composition was: 5% lactose, 1.5 % soy peptone, 1% yeast extract, 1 mM MgSO_4 _and 0.1 mM MnSO_4_.

In a series of 58 1-L fermentations the following parameters were tested and optimized: pH, neutralization agent, addition of phosphate and zinc, fermentation temperature, the time point of induction, i.e. cell density, the concentration of peptone, yeast extract and lactose in the medium and the lysostaphin production time after induction.

### pH and neutralizing agent

**Figure 2:**
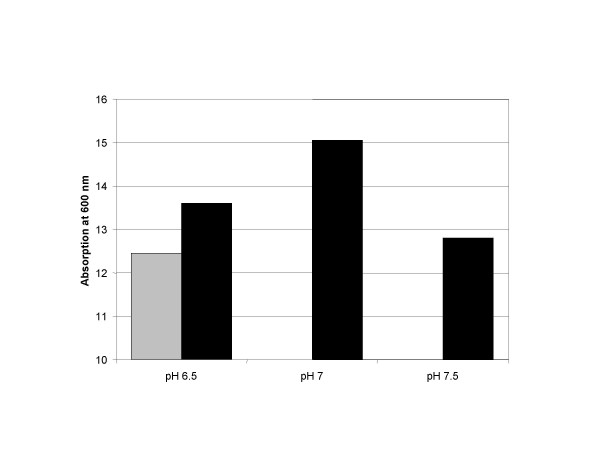
**Biomass production depending on pH and neutralizing agent. **The grey bar indicates the use of NaOH for neutralization of the culture and black bars indicate the use of NH_4_OH for neutralization. Biomass production is expressed in optical density at 600 nm (path length = 1 cm) (the dry cell weight factor is 0.3 g/L/1 OD_600_). Higher biomass production may be beneficial for higher yield of the produced heterologous protein.

The main fermentation end product of *L. lactis *is lactate, which is toxic and retards growth above a certain concentration. The toxic effect of lactate depends on its undissociated form [21]. Therefore, any increase of the pH can lead to a prolongation of growth in the presence of higher concentrations of lactate. NaOH is a more alkaline neutralizing agent than NH_4_OH and could cause cell damage. Furthermore, NH_4_OH could contribute to the ammonium metabolism of the cell [22]. Figure 2 shows that neutralization with NH_4_OH has a small effect, however the combination of NH_4_OH with an increase of the fermentation pH to 7.0 leads to prolonged exponential growth (not shown) and to a higher final cell density. Increase of the pH to 7.5 leads to a retardation of the growth rate and has therefore not been further pursued.

### Addition of phosphate and zinc

**Figure 3:**
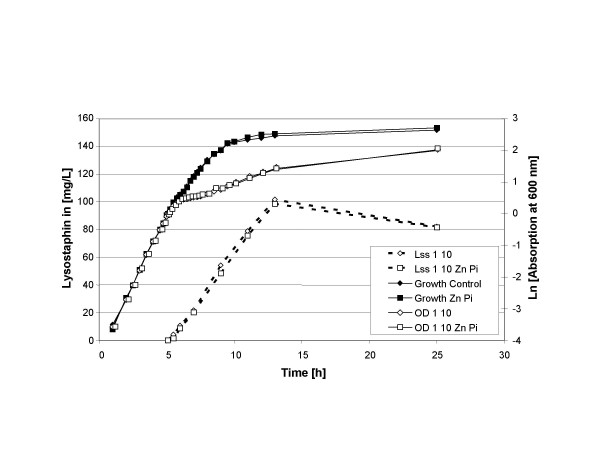
**Influence of zinc and phosphorus (Zn P_i_) on growth, induction and lysostaphin production. **Growth of the culture is indicated with continuous lines. Production of lysostaphin is indicated with broken lines. The cells are induced for lysostaphin (Lss) production at OD_600 _= 1 with 10 ng/mL nisin. [OD 1 10] or [Lss 1 10] indicate conditions for growth or lysostaphin production.

0.01% of sodium phosphate (Na_2_HPO_4 _*2 H_2_O) was added to evaluate the effect on growth performance. Furthermore, zinc was specifically added, because lysostaphin is a metallo-enzyme with zinc as co-factor [16]. Elemental analysis of the basic medium had shown that it contained 1.2 mg/L Zn^2+^. This amount would be sufficient for a maximum of 500 mg/L lysostaphin if all the zinc were available. Therefore, 100 μM (16 mg/L) ZnSO_4 _was added, which would allow the production of a maximum of 2.5 g/L active lysostaphin. Addition of these components had no significant effect on growth under the initial fermentation and induction conditions (Figure 3). However, in a later stage when induction was performed at a higher cell density and with more nisin (induction at OD_600 _= 5 with 40 ng/ml nisin) considerably less lysostaphin was formed when phosphate was omitted (150 mg/L without phosphate versus 220 mg/L lysostaphin with phosphate).

After these initial experiments, it was decided to set the following basic fermentation conditions: pH at 7.0, to use NH_4_OH as neutralizing agent, to add both 0.01 g/L sodium phosphate (Na_2_HPO_4 _*2 H_2_O) and 100 μM ZnSO_4_. To establish a baseline for lysostaphin production and to analyze the effect of phosphate or zinc on lysostaphin production, an induction experiment was carried out. Induction of lysostaphin production was initiated at OD_600 _= 1.0 with 10 ng/mL nisin. Figure 3 shows the basic pattern of growth and induction. After the addition of nisin, product formation begins immediately, and occurs parallel to growth. When lysostaphin production was induced, growth of the culture slowed down considerably about 30 min after induction. This is accompanied by a steep drop of the viable counts on lactose M17 agar plates of 4 orders of magnitude. However, despite the growth retardation, lysostaphin production was linear for 8 h before it abruptly ended (Figure 3) [15].

### Fermentation temperature

**Figure 4:**
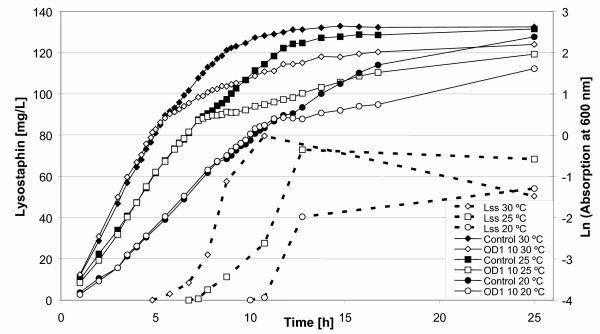
**Influence of the temperature on growth, induction and lysostaphin production. **Growth of the culture is indicated with continuous lines. Production of lysostaphin is indicated with broken lines. The cells are induced at the different growth temperatures for lysostaphin production at OD_600 _= 1 with 10 ng/mL nisin. [Lss 30°C], condition for lysostaphin production; [OD 1 10 30°C], condition for growth.

Overproduction of proteins can trigger stress responses and thus e.g. degradation of the target protein in the cell [23]. One of the strategies employed to prevent or minimize this effect is to lower the fermentation temperature and thereby lower the threshold for the induction of the stress response, and also slow down protein production. Figure 4 shows both growth and lysostaphin production during fermentation at 30°C, 25°C and 20°C. Induction was carried out at a cell density of OD_600 _= 1 (0.3 g/L cell dry weight) with 10 ng/mL nisin. At 25°C and 20°C growth is, as expected, slower than at 30°C. Also lysostaphin production is slower and much lower at 20°C as compared to 30°C. However, these experiments also show that the NICE system works at these temperatures; especially at 25°C similar yields are obtained as at 30°C. However, because of the faster production and the slightly higher yields, 30°C was chosen as fermentation temperature for all subsequent experiments.

### Combination of cell density at induction and the amount of added nisin

**Figure 5:**
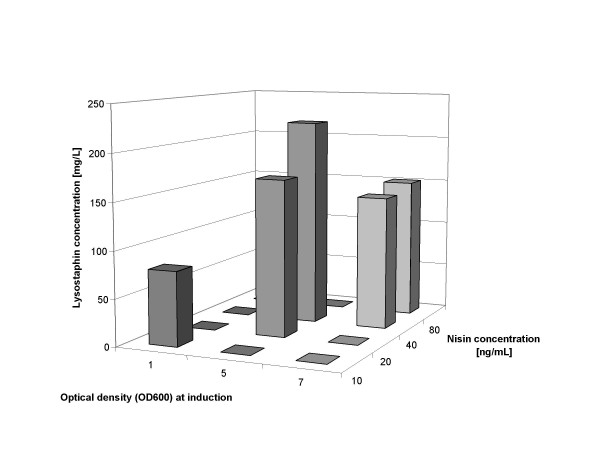
**Influence of the time point of induction and the amount of added nisin on the amount of produced lysostaphin 8 hours after induction. **The time point of induction is indicated as the optical density of the culture [OD_600_] at which nisin was added.

If induction could be performed at higher cell densities a higher yield per volume of culture fluid could be expected. Since at higher cell densities more nisin may also be needed for complete induction, these parameters were tested together. Initial experiments showed that induction could be performed at an OD_600 _of 3 or even 5. Figure 5 shows an experiment were induction was tested at OD_600 _values of 1, 5 and 7 (equal to a cell dry weight of 0.3 g/L, 1.5 g/L and 2.1 g/L, respectively) with nisin concentrations of up to 80 ng/mL. Clearly lysostaphin production can be increased when the culture is induced at higher cell densities. At the same time, more nisin needs to be added for maximum induction. When induction was performed at OD_600 _= 5, 160 mg/L lysostaphin was formed with 20 ng/mL nisin and 220 mg/L lysostaphin was produced when 40 ng/mL nisin was used for induction. This means that there was a clear correlation between the cell density at induction and the amount of nisin that is needed for maximal induction. Induction at even higher cell densities, OD_600 _= 7 with 40 and 80 ng/mL nisin, was also tried; however this did not lead to higher lysostaphin yields, indicating that there is a limit in the growth cycle beyond which induction becomes ineffective. Exponential growth without induction continues until about OD_600 _= 8, after which growth slows down as the culture slowly approaches the final cell density of about 15. This is due to the accumulating lactate that increasingly interferes with the energy metabolism of the cell [24,25]. The same mechanism also affects and limits the production of the recombinant protein. Induction at a cell density of OD_600 _= 5 with 40 ng/mL nisin resulted in a maximum yield of the recombinant protein.

### Addition of extra nutrients

**Figure 6:**
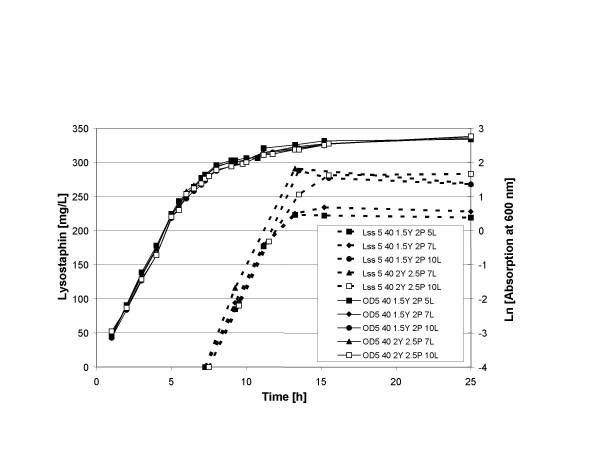
**Influence of the medium composition on lysostaphin yield. **Growth of the culture is indicated with continuous lines. Production of lysostaphin is indicated with broken lines. Lysostaphin production was induced at OD_600 _= 5 with 40 ng/mL nisin. Y, P, and L indicate the percentage of yeast extract, peptone and lactose, respectively, used in the culture medium. [Lss 5 40 1.5Y 2P 5L], conditions for lysostaphin production; [OD 5 40 1.5Y 2P 5L], conditions for growth.

**Figure 7:**
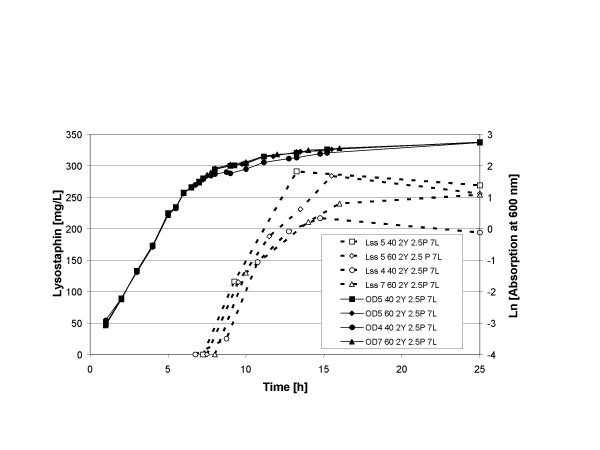
**Influence of induction conditions and the medium composition on lysostaphin yields. **Growth of the culture is indicated with continuous lines. Production of lysostaphin is indicated with broken lines. The cultures were induced at an O_600 _= 4, 5 or 6 with 40 or 60 ng/mL nisin. Y, P, and L indicate the amount in % of yeast extract, peptone and lactose, respectively, used in the culture medium. [Lss 5 40 2Y 2.5P 7L], conditions for lysostaphin production; [OD 5 40 2Y 2.5P 7L], conditions for growth.

Although induction was successful at a five times higher cell density than the original induction conditions, we observed only about 2 – 2.5 times more product formation. This could be due to either a shortage in building blocks, i.e. amino acids or a limitation of the energy supply, i.e. the fermentable sugar. Therefore, the next step was to supply more nitrogen and carbohydrate sources. Initial experiments showed that the lysostaphin yield increases when either more yeast extract or more peptone was added and increases even more when both nutrients were increased simultaneously (results not shown). Figure 6 shows a series of fermentations in which the influence of a combination of nitrogen and carbon sources on product formation was investigated. Lysostaphin production was induced at OD_600 _= 5 (1.5 g/L cell dry weight) with 40 ng/mL nisin. The addition of extra carbon source, 7% lactose versus 5% lactose, did not significantly increase the production of lysostaphin. However, doubling of the carbon source did raise lysostaphin yield from 225 mg/L to 290 mg/L. An increase in the supply of the nitrogen sources peptone and yeast extract also raised the lysostaphin yield form 225 mg/L to 290 mg/L. In a final experiment we used the best medium (2.5% peptone, 2% yeast extract and 7% lactose) and looked again at the combination of cell density and the amount of nisin for induction (Figure 7). Clearly, either lower or higher cell densities (OD_600 _= 4 or 7) lead to slower production and lower yields. The same is true for the addition of more nisin (60 ng/mL), which at that concentration probably has a detrimental effect on the production of lysostaphin.

### Summary of the optimization

The highest production of lysostaphin was observed under the following conditions: the pH is fixed at 7.0, the temperature is 30°C, the neutralizing agent is NH_4_OH, medium components are 7% lactose, 2.5% peptone, 2% yeast extract, 0.01% sodium phosphate (Na_2_PO_4 _*2 H_2_O), 100 μM ZnSO_4_, 1 mM MgSO_4 _and 0.1 mM MnSO_4_, cells are grown to an OD_600 _= 5 and induced with 40 ng/mL nisin. Lysostaphin production proceeded for 6 to 8 hours and then abruptly stopped. After that time there may be a small decrease of lysostaphin concentration over time. However, this depended on the medium composition and the fermentation conditions (Figures 4, 6 and 7).

## Discussion

The nisin-controlled gene expression system NICE is widely used for a multitude of different applications. However, induction of this gene expression system has never been optimized for either laboratory conditions or for industrial-scale applications. In the present publication we show that the yield of the production of a heterologous protein can be increased at least three-fold by careful optimization of the fermentation and induction conditions. A number of general observations have been made:

(I) in a pH controlled culture, the point at which the pH is fixed and the neutralizing agent influence the general growth and biomass yield of the culture. A neutral pH and a mild neutralizing agent such as NH_4_OH was beneficial for growth of lactococci.

(II) Nisin induction works over a broad range of temperatures, even at 20°C. However, there is a clear correlation between the temperature of the fermentation and the speed and yield of the induction. The lower the temperature, the slower was the response to nisin and the lower was the yield of the heterologous protein. For lysostaphin production, a temperature of 30°C was found to be the optimum. Figure 4 may give a hint that it is worthwhile to also consider production at a lower temperature. At 25°C maximum lysostaphin production was slower and somewhat lower than at 30°C; however it seemed to be more stable in the cells, as can be seen at the end of the culture.

(III) Addition of extra and specific nutrients may be needed. Lysostaphin is a zinc-containing enzyme. Production of large amounts of active enzyme may be limited by the amount of zinc that is present in the different medium components. Therefore, extra zinc was added.

More generally, phosphate is needed for DNA biosynthesis and the energy metabolism of the cell. There are certain amounts of phosphorus compounds in the yeast extract and in the peptone, but growth at higher cell densities and product formation may require more phosphorus. Initially, little difference was seen after addition of phosphate, however, in an induction experiment at higher cell densities, strongly reduced product formation was observed (150 mg/L lysostaphin without extra phosphate versus 220 mg/L with phosphate). Zinc is an example of specific components that need to be selected and optimized for each target protein individually.

(IV) There is a strong correlation between the medium composition, the cell density of the culture at the moment of induction and the amount of nisin that is added. Induction is a dynamic process and needs growing cells both for the induction to occur and for the subsequent protein production to proceed as fast and as long as possible. In Figure 5 we saw increases in lysostaphin production after both the cell density for induction and the amount of nisin were increased. However, when the induction occurs too late in the growth cycle (e.g. OD_600 _= 7) no further product increase can be observed, even when more nisin was added. Similarly, if too much nisin is added (see Figure 7) it will become detrimental for product formation. Under the present conditions the maximum yield was reached at an OD_600 _around 5 with about 40 ng/mL nisin. These parameters can directly be used for the increased production of any other heterologous protein and as a starting point for further optimization of the respective process.

(V) As can be seen in Figure 6, lysostaphin production was still limited by the supply of nutrients in the fermentation medium. Addition of higher amounts of peptone, yeast extract and sugar significantly increased the production of lysostaphin. These parameters need to be carefully optimized, since they make up the highest costs of the growth medium.

The present publication outlines the optimization of controlled expression of heterologous genes in *L. lactis *using the NICE system. Careful optimization of a number of key parameters leads to a considerable increase in the overall yield of the target protein. The current outline gives a framework for this optimization. However, since every protein is different, a number of steps need to be checked and fine-tuned individually.

After the present round of optimization, the model protein lysostaphin could be produced up to 300 mg/L. Since lysostaphin has a growth-inhibiting effect on the host cells, and maybe also on its own production, a higher production capacity of the *L. lactis *host can be inferred for target proteins that do not have functionally detrimental effects on the host cells. Application of the described optimization to other target proteins will show the actual potential and limits of *L. lactis *for the industrial production of heterologous proteins.

## Conclusion

The nisin-controlled gene expression system NICE of *Lactococcus lactis *is one of the most widely used Gram-positive gene expression systems. To date, no systematic study has been undertaken to optimize the system for maximum yields, especially for industrial scale applications. The present study shows that by careful optimization of growth, induction and target protein-specific parameters, an at least three-fold increase of the yield can be achieved.

## Methods

### Bacterial strains and growth media

*Lactococcus lactis *subsp. *cremoris *NZ3900 [26] carrying plasmid pNZ1710 (plasmid with gene for mature lysostaphin under control of the *nisA *promoter; [15]) was stored as frozen stock in M17 medium [27] containing 0.5% lactose as carbon source and plasmid selection agent. The basic fermentation medium contained 5% lactose (Lactochem, Borculo Domo Ingredients, Zwolle, The Netherlands), 1.0% yeast extract (Biospringer, Maisons-Alfort, France), 1.5% soy peptone (Merck, VWR International, Amsterdam, The Netherlands), 1 mM MgSO_4 _and 0.1 mM MnSO_4_. All components were dissolved in water and sterilized at 110°C for 20 min. Additional components such as Na_2_HPO_4 _and ZnSO_4 _were filter sterilized and added separately.

Nisin for induction was prepared as follows: 0.04% nisin powder (Sigma-Aldrich Chemie, Zwijndrecht, The Netherlands) was dissolved in 0.05% acetic acid and precipitated proteins were removed by centrifugation. Nisin was added for induction as indicated in the Results section.

### Fermentations

The strain was taken from stock and sub-cultured twice before inoculation. 1-L Applicon fermenters were used coupled to an Applicon Biocontroller ADI 1030 (Applicon, Frederiksberg, Denmark) for pH and temperature control. The pH of the culture was controlled at the indicated values (see Results) with either 2.5 M NaOH or 2.5 M NH_4_OH. The cell density was measured in samples that were taken at regular intervals, by determining the absorbance at 600 nm with a path length of 1 cm [OD_600_]. For lysostaphin measurements, cells of appropriate samples were sedimented by centrifugation and stored at -20°C. After thawing, cell density was adjusted to OD_600 _= 10 (according to the initial cell density reading) and 1 ml of the cell suspension was mixed with 1 g glass beads (0.1 mm Zirconia/Silica beads from Biospec products, Bartlesville, OK., U.S.A.) in screw-cap Eppendorf tubes. Subsequently, cells were subjected to bead-beating in a FastPrep beadbeater (FP120, QBiogene, Irivne, CA, U.S.A.), adjustment 4, 4 × 30 s. After the beads were allowed to sediment, lysostaphin concentration was determined in the whole cell extract.

### Lysostaphin measurement

Lysostaphin was quantified using SDS-capillary zone electrophoresis (SDS-CZE). The SDS sample buffer was prepared by dissolving in ca. 80 mL of water 606 mg of tris(hydroxymethyl)aminomethane (Tris), 1.00 g of sodium dodecylsulphate (SDS) and 37 mg of EDTA. Hydrochloric acid (0.1 M, 14.7 mL) was added and the solution was made up to 100 mL. Daily, 25 mg of DTT was added to 10 mL SDS sample buffer. This solution was used to dissolve cell extract samples (see below).

The SDS-CZE separation was performed using a Beckman Coulter P/ACE MDQ capillary electrophoresis system (CA, U.S.A.), equipped with a UV-detector operating at 214 nm using a 30 cm coated capillary and the separation buffer of the Beckman Coulter SDS 14–200 kit. The capillary temperature was set at 20°C.

Before each analysis, the 30 cm capillary was rinsed in the reverse direction for 1 min at 20 psi using 0.1 M HCl and subsequently for 3 min at 20 psi with SDS separation buffer. The sample solution was injected for 30 s at 1 psi, followed by forward rinse for 30 s at 0.5 psi of SDS sample buffer/water (1:1). The separation voltage was ramped in 1 min to 9 kV (ground at detector outlet) and held constant for 18 min.

A standard solution of lysostaphin was prepared as follows. From a solution of a known concentration of lysostaphin (ca. 1 mg/mL) 40 μL was pipetted into an Eppendorf vial of 0.5 mL and 120 μL of SDS sample buffer was added. The closed vial was incubated for 30 min at 80°C and subsequently rapidly cooled using ice water. From this solution 80 μL was transferred to the sample vial (of 0.2 mL Eppendorf vial).

For the preparation of samples of cell extracts, the same procedure as that for the standard solution of lysostaphin was followed, except that after cooling in ice water the solution was centrifuged for 5 min at 3000 g to remove any possible traces of precipitated proteins.

### Element analysis

Analysis of minerals and trace elements in the basic medium was carried out by ICP-AES (Inductively Coupled Plasma – Atomic Emission Spectrometry) using the Vista Axial ICP of Varian (Palo Alto, CA, U.S.A.). The growth medium sample was prepared for ICP by dry-ashing, dissolved in nitric acid and measured against standards of Ca, Mg, Na, K, P, Fe, Zn, Mn and Cu.

## Authors' contributions

IM and JM were the initiators and main supervisors of the experiments. KO developed the CE lysostaphin assay. IM and ES developed the optimization strategy.
